# Alternative Splicing of L-type Ca_V_1.2 Calcium Channels: Implications in Cardiovascular Diseases

**DOI:** 10.3390/genes8120344

**Published:** 2017-11-24

**Authors:** Zhenyu Hu, Mui Cheng Liang, Tuck Wah Soong

**Affiliations:** 1 Department of Physiology, Yong Loo Lin School of Medicine, National University of Singapore, Singapore 117593, Singapore; phshz@nus.edu.sg (Z.H.); phslmc@nus.edu.sg (M.C.L.); 2 Neurobiology/Ageing Programme, Center for Life Sciences, NUS Graduate School for Integrative Sciences and Engineering, Singapore 117456, Singapore; 3 Neurobiology/Ageing Programme, Center for Life Sciences, National University of Singapore, Singapore 117456, Singapore

**Keywords:** L-type Ca_V_1.2 calcium channel, alternative splicing, cardiovascular diseases

## Abstract

L-type Ca_V_1.2 calcium channels are the major pathway for Ca^2+^ influx to initiate the contraction of smooth and cardiac muscles. Alteration of Ca_V_1.2 channel function has been implicated in multiple cardiovascular diseases, such as hypertension and cardiac hypertrophy. Alternative splicing is a post-transcriptional mechanism that expands Ca_V_1.2 channel structures to modify function, pharmacological and biophysical property such as calcium/voltage-dependent inactivation (C/VDI), or to influence its post-translational modulation by interacting proteins such as Galectin-1. Alternative splicing has generated functionally diverse Ca_V_1.2 isoforms that can be developmentally regulated in the heart, or under pathophysiological conditions such as in heart failure. More importantly, alternative splicing of certain exons of Ca_V_1.2 has been reported to be regulated by splicing factors such as RNA-binding Fox-1 homolog 1/2 (Rbfox 1/2), polypyrimidine tract-binding protein (PTBP1) and RNA-binding motif protein 20 (RBM20). Understanding how Ca_V_1.2 channel function is remodelled in disease will provide better information to guide the development of more targeted approaches to discover therapeutic agents for cardiovascular diseases.

## 1. Introduction

L-type voltage-gated calcium channels (LTCC) contain four subtypes, Ca_V_1.1, Ca_V_1.2, Ca_V_1.3 and Ca_V_1.4, and they are sensitive to blockade by 1,4-dihydropyridines (DHPs) [1]. Among the L-type calcium channels, Ca_V_1.1 channels are mainly localized in skeletal muscle, while Ca_V_1.2 channels predominate in brain [2], cardiac [3] and vascular smooth muscle [4]. On the other hand, Ca_V_1.3 channels are mainly expressed in the adrenal gland [5], pancreas [6], brain [7], cochlea [8] and sinoatrial node [9], and are essential for neurotransmission in the auditory hair cells and for cardiac pacemaker activity [10]. The Ca_V_1.4 channels, however, have a restricted expression and play an important role in synaptic transmission in the retina [11].

**Figure 1:**
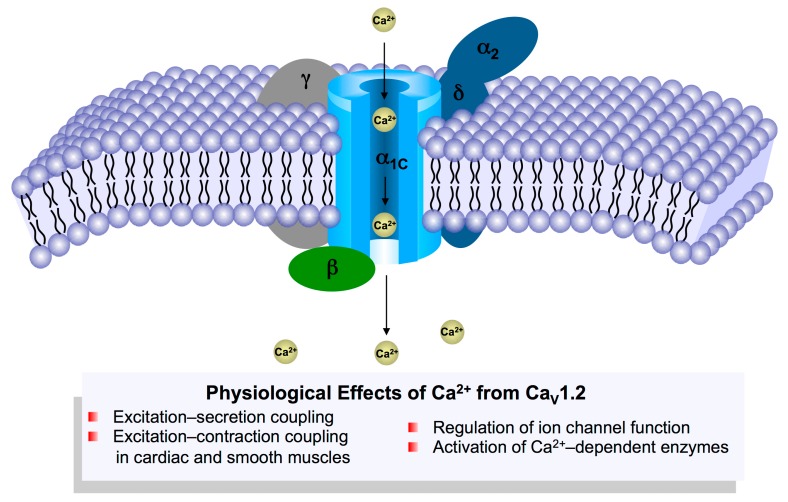
Subunit assembly of the Ca_V_1.2 protein complex and the major physiological effects of Ca^2+^ entry into the muscle cells through Ca_V_1.2 channels.

The Ca_V_1.2 channel is a multi-protein complex and is composed of the transmembrane pore-forming α_1C_ subunit (referred to as the Ca_V_1.2 channel in this review), the auxiliary α_2_δ and β subunits, and also the γ subunit in skeletal muscle (Figure 1) [12]. The α_2_δ-subunit has four isoforms (α_2_δ_1–4_) and the smooth muscle isoform, α_2_δ_1_, has been shown to promote the surface expression of Ca_V_1.2 channels and increase the contractile ability of cerebral arteries [13,14]. Moreover, the β-subunit is essential for Ca_V_1.2 channel function, as binding of the β-subunit to the α_1_-interacting domain (AID) within the I–II loop prevents endoplasmic reticulum-associated degradation (ERAD), and promotes channel trafficking to the cell membrane [15]. Similar to the α_2_δ-subunit, the β-subunit also has four subtypes (β_1–4_). In smooth muscle, the β_2_ and β_3_ subunits were dominantly expressed with detectable protein levels [16]. Global β_2_ knockout was reported to be embryonic lethal due to cardiovascular dysfunction [17], while β_3_-deficient mice infused with angiotensin II displayed inhibited Ca_V_1.2 channel expression and lower blood pressure compared with wild-type mice [18]. In cardiac muscle, 18 different β-subunit isoforms have been identified in canine and human ventricle with distinct subcellular localizations [19]. The β_2_ subunit was suggested to be the predominant isoform in the heart [19]. Moreover, overexpression of β_2a_-subunit in mice resulted in higher L-type calcium currents and cardiac hypertrophy at 4 months of age [20].

Alternative splicing is a key biological mechanism that generates functionally distinct splice variants of Ca_V_1.2 channels, some of which may be tissue-specific [21], developmentally regulated [22] or involved in cardiovascular diseases, such as atherosclerosis [23] and heart failure [24]. The human Ca_V_1.2 gene (*CACNA1C*) consists of 50 exons, while that from mouse and rat have 49 exons due to the lack of exon 45. Among these exons, at least 20 of them in the N- and C- terminus, loops I–II and II–III, S6 of Domain I, S2 of Domain II, S3 of Domain IV, between S5 and S6 in Domain I, between S2 and S3 in Domain IV and between S3 and S4 in Domain IV undergo alternative splicing [25] (Figure 1). More than 50 Ca_V_1.2 splice combinations have been identified in the heart and smooth muscles, with different localizations and functions [24].

In this review, we have mainly focused on the alternative splicing of Ca_V_1.2 channels that is developmentally regulated or cardiovascular disease-related by highlighting splice variant-specific biophysical properties and functions. Moreover, we will briefly introduce the splicing factors that regulate the alternative splicing of Ca_V_1.2 channels.

## 2. Overview of Ca_V_1.2 Channel Function in the Cardiovascular System

**Figure 2:**
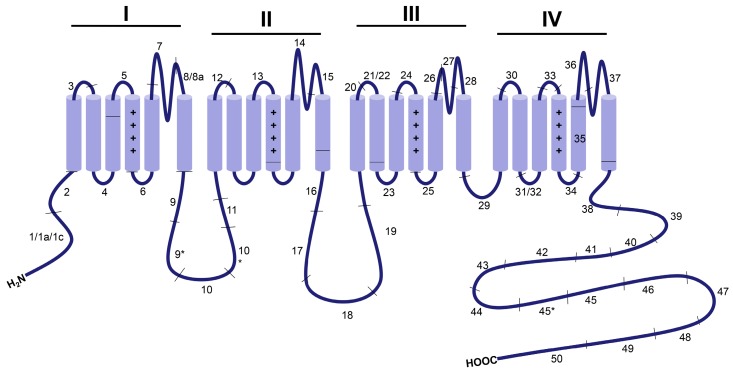
Representative sites of alternative splicing in the pore-forming Ca_V_α_1C_ subunit. The exons encoding α_1C_-subunit are indicated numerically and separated by lines across the schematic diagram.

Calcium ion influx through Ca_V_1.2 channels is critical for the initiation of various cell processes including excitation–contraction coupling via Ca^2+^-induced Ca^2+^ release [26], excitation–secretion coupling [27], regulation of Ca^2+^-dependent enzymes and modulation of the biophysical properties of ion channels [12] (Figure 2). Alterations in vascular and cardiac Ca_V_1.2 calcium channel activity have been associated with hypertension, cardiac hypertrophy and heart failure [28]. Smooth muscle-specific knockout of Ca_V_1.2 channels in mice abolished the development of myogenic tone and drastically reduced arterial blood pressure [29], validating the central role Ca_V_1.2 channels play in regulating blood pressure. Moreover, the Ca_V_1.2 protein level was also significantly up-regulated in the mesenteric and skeletal artery of spontaneously hypertensive rats (SHR) [30]. Additionally, malfunction in Ca_V_1.2 channels is associated with cardiac disorders including Timothy syndrome that is characterized by a long QT interval and ventricular arrhythmia due to sustained activation of Ca_V_1.2 channels, and Brugada syndrome that is notable for a short QT interval and sudden cardiac death due to inactivation of Ca_V_1.2 channels [31]. While the role of Ca_V_1.2 channels in electrical heart diseases is well known, its role in mechanical or structural heart diseases remains controversial. Cardiac-specific overexpression of β_2a_-subunit or α_1C_-subunit in mice was reported to induce cardiac hypertrophy and cardiomyopathy by increasing Ca^2+^ influx through Ca_V_1.2 channels [20,32]. However, α_1C_^+/−^ mice with decrease in Ca^2+^ influx through Ca_V_1.2 channels displayed a similar phenotype [28]. The reason for Ca_V_1.2 downregulation-induced cardiac hypertrophy may be that, due to the lack of sufficient LTCC current, Ca^2+^ release from Ryanodine receptor 2 (RYR2) is sensitized to compensate for reduced systolic Ca^2+^ in order to maintain cardiac contractility, thereby resulting in hypertrophic remodeling through activating the calcineurin/Nuclear factor of activated T cells (NFAT) pathway. However, this notion still requires more experiments for validation as the Ca_V_1.2 channels from two different microdomains in human and feline cardiomyocytes were considered to play different roles in cardiac function [33,34]: One sub-population of Ca_V_1.2 channels in the T-tubules accounts for excitation–contraction coupling, while another in the caveolae activates the transcription factor NFAT by hypertrophic Ca^2+^ signaling. In contrast, with specific overexpression of a caveolae-targeted Ca_V_1.2 inhibitor (CBD-REM, a truncated REM^1-265^ fused N-terminal to caveolin-binding domain) or activator (CBD-β_2a_, a mutated β_2a_^C3S/C4S^ fused C-terminal to CBD) in cardiac muscles, the transgenic mice subject to the transverse aortic constriction model did not show significant changes in hypertrophic signaling and cardiac function [35], suggesting that, at least in adult mouse heart, the caveolae-resident Ca_V_1.2 channels may not contribute to the development of cardiac hypertrophy. However, this study does not exclude the possibility that caveolae-resident LTCC could moderately affect the predisposition of the mouse heart to a lower-level stress, as β_2a_-trangenic mice displayed more severe cardiac hypertrophy under phenylephrine stimulation through Calcium/calmodulin-dependent protein kinase II (CaMKII)-mediated phosphorylation of caveolae-resident β_2a_ subunits and the resulting up-regulation of caveolae-resident LTCC [36].

## 3. Developmentally Regulated Cardiac Ca_V_1.2 Splicing

**Figure 3:**
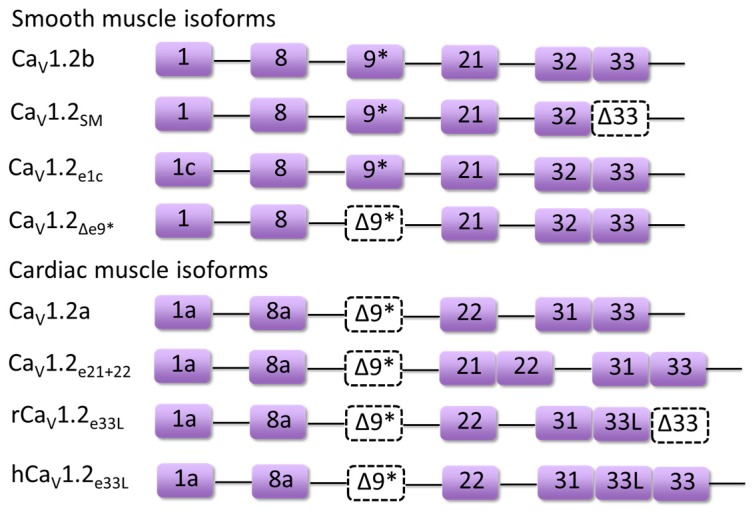
Combinatorial key alternatively spliced exons of major Ca_V_1.2 isoforms mentioned in this review such as Ca_V_1.2a, Ca_V_1.2b, Ca_V_1.2_e21+22_ are labeled as purple boxes. Dashed boxes indicate the exclusion of exons (Δ9* or Δ33).

During cardiac development, the inclusion level of certain alternatively spliced exons in Ca_V_1.2 channels has been shown to change gradually in rodent and human hearts, suggesting a differential role of Ca_V_1.2 isoforms in fetal and mature hearts. One of the developmentally regulated Ca_V_1.2 splice variants is the novel Ca_V_1.2_33L_ channel containing exon 33L that forms part of the S3–4 linker of domain IV (Figure 3). The inclusion of rat exon 33L (RGSC 6.0/rn6, chr4: 150674656-150674726) causes a frame shift, which results in premature termination and C-terminal truncation of the channel protein to produce a non-functional Ca_V_1.2 channel [22]. The percentage of this splice variant was found to reduce from 9.7% in neonatal hearts to 4.3% in left ventricles of adult hearts. More importantly, this non-functional Ca_V_1.2 splice isoform showed significant dominant-negative suppression on wild-type Ca_V_1.2 channel function by accelerating the degradation of Ca_V_1.2 channels via the ubiquitin-proteasome system in transfected HEK 293 cells. However, the alternative splicing in exon 33L was found to be species-specific, as human exon 33L (GRCh38/hg38, chr12: 2648403-2648474) with a single nucleotide insertion generated a functional full-length Ca_V_1.2 channel (Figure 3) which can conduct Ca^2+^ current, although at a much lower level than wild-type human Ca_V_1.2 channels.

Besides exon 33L, there is another alternatively spliced site in the S3–4 linker of domain IV, namely mutually exclusive exons 31 (RGSC 6.0/rn6, chr4: 150682589-150682672) and exon 32 (RGSC 6.0/rn6, chr4: 150681903-150681986), which are also developmentally regulated in rat heart [37]. Both exons 31 and 32 were reported to be equally expressed in newborn and fetal rat hearts, but only exon 31 was significantly reduced in adult hearts, which indicated that a significant switch from exon 31 to 32 occurred during cardiac development. To date, it still remains unclear what specific physiological role each Ca_V_1.2 isoform may play during cardiac development. Whole-cell patch-clamp recordings of exon 31- or exon 32-contaning human Ca_V_1.2 channels in transfected HEK 293 cells did not show any changes in their electrophysiological properties [38]. However, the biophysical properties of exon 31- or exon 32-containing cardiac Ca_V_1.2 channels need further validation in native fetal or adult cardiomyocytes and as such their defining roles in cardiac development remain to be examined.

Recently, a new developmentally regulated Ca_V_1.2 splice variant in rodent and human hearts, Ca_V_1.2_e21+22_ (Figure 3) was identified [39]. The Ca_V_1.2_e21+22_ channel contains both exon 21 and exon 22. Exons 21/22 are mutually exclusive exons and the exon encodes the IIIS2 transmembrane segment and part of the linker region between IIIS1 and IIIS2. Transcript-screening showed that the abundance of exons 21 + 22 inclusion (RGSC 6.0/rn6, chr4: 150718378-150718639) was reduced from 14.3% in rat neonatal heart to 5.5% in adult heart. Further functional assays showed that the Ca_V_1.2_e21+22_ channel was not able to conduct any Ca^2+^ influx, but had a stronger interaction with β subunits. Thus, co-expression of Ca_V_1.2_e21+22_ channels was able to promote the proteasomal degradation of wild-type Ca_V_1.2 channels by competing for β-subunits.

## 4. Cardiovascular Conditions-Related Splice Variants of Ca_V_1.2 Channels

### 4.1. Timothy Syndrome

Exon 8 (GRCh38/hg38, chr12: 2504842-2504945)-containing Ca_V_1.2 splice exhibited higher sensitivity to dihydropyridine (DHP), which may be the reason why the smooth muscle splice variant Cav1.2b (Figure 3) expressing exon 8 is more sensitive to inhibition by DHPs than the heart variant Cav1.2a (Figure 3) containing exon 8a (GRCh38/hg38, chr12: 2504436-2504539) [25]. Mutations found in the mutually exclusive exons 8 and 8a of the human *CACNA1C* gene that encodes the Ca_V_1.2 channel are associated with the multi-organ disorder named Timothy syndrome (TS) [31,40]. A point to note is that the authors of the TS articles have labelled exons 8 and 8a differently from the rest of the community. Exon 8a, which is upstream of exon 8 in genomic sequence and known to be predominantly expressed in cardiac muscle [41], is labelled as exon 8 by the authors of the TS articles [31]. Similarly, exon 8, which is predominantly expressed in smooth muscles [41], is labelled as exon 8a by the authors [31]. Their nomenclature for exon 8 and 8a is still used for TS in this review to avoid confusion when referencing the original articles. One de novo missense mutation G406R in exon 8 (GRCh38/hg38, chr12: 2504436-2504539) or exon 8a (GRCh38/hg38, chr12: 2504842-2504945) has been reported to induce classical Timothy syndrome (TS1) and the mutant channels lack normal voltage-dependent inactivation (VDI) which led to sustained depolarization; while G402S and G406R mutations in exon 8 caused more severe atypical Timothy syndrome (TS2), which has been shown to generate long QT syndrome and resultant arrhythmia. In addition to defects in VDI, the TS variants also led to calcium-dependent inactivation (CDI) deficits with G402S mutation causing a decrease in *F*_CDI_ (a function of Ca^2+^) and G406R mutation, primarily resulting in a reduction of *CDI*_max_ (a function of channel gating) [42]. As exon 8 (GRCh38/hg38, chr12: 2504436-2504539) is much more dominant in the heart than exon 8a (GRCh38/hg38, chr12: 2504842-2504945), generally patients suffering from atypical TS displayed worsened cardiac defects.

In addition, another six novel gain-of-function mutations, A28T, R860G, I1166T, I1166V, I1475M and E1496K identified in patients with long QT, which resulted in a similar strong gain-of-function as the known TS mutations, did not cause TS, but only caused the non-syndromic long QT [43]. These results suggested that TS’s phenotypes may only be produced by specific Cav1.2 mutations located in exon 8/8a. As for those gain-of-function mutations in other exons of Cav1.2, they may only lead to restricted phenotypes such as long QT syndrome without the multi-organ characteristics of TS. However, these six mutations were identified in blood lymphocytes from patients. Therefore, it remains unclear whether these mutant Ca_V_1.2 channels are expressed in the hearts of the patients with non-syndromic long QT syndrome or even with TS.

### 4.2. Heart Failure

Ca_V_1.2 channel lacking exon 33 (Ca_V_1.2_Δe33_) showed hyperpolarized shifts for steady-state inactivation and activation potential compared to the Ca_V_1.2_e33_ channel in transfected HEK 293 cells [44]. Moreover, the exclusion level of exon 33 (RGSC 6.0/rn6, chr4: 150674623-150674655) was found to increase in the scar region of rat heart subject to chronic myocardial infarction [44]. However, it is still not clear how this molecular alteration of exon 33 affects cardiac function. In exon 33 (GRCm38/mm10, chr6: 118630399-118630431) null mice, the cardiac contractility and output is significantly increased and the hearts were more susceptible to the generation of ventricular tachyarrhythmia [45]. Additionally, the inclusion level of exon 33 (GRCh38/hg38, chr12: 2648475-2648507) increased significantly by about 20% in failing hearts from patients with ischemic or dilated cardiomyopathy [45]. However, the potential role that exon 33 inclusion in Ca_V_1.2 channels may play in the pathogenesis of human heart failure remains unclear.

Besides exon 33, the mutually exclusive exons 31/32 were also associated with human heart failure [24]. Briefly, the level of exon 32 inclusion (GRCh38/hg38, chr12: 2634297-2634380) is about two times higher than exon 31 (GRCh38/hg38, chr12: 2633629-2633712) in left ventricles of human failing hearts, while the exon 31 level is 2.5 times higher in non-failing hearts. However, the regulatory mechanisms underlying isoform switching are still unknown and the specific physiological role each Ca_V_1.2 isoform may play in human normal and failing hearts remains to be determined.

### 4.3. Atherosclerosis

Atherosclerosis is characterized by inflammation-mediated endothelial perturbation, local release of cytokines and proliferation and migration of smooth muscle cells in medium and large size arteries [46]. In atherosclerotic smooth muscle cells isolated from carotid and femoral arteries of patients with atherosclerosis, the switch of exon 21 (GRCh38/hg38, chr12: 2597230-2597289) to exon 22 (GRCh38/hg38, chr12: 2597437-2597496) is the molecular signature of *CACNA1C* alternative splicing [47]. It is noteworthy that, in the quiescent non-proliferating smooth muscle cells, the A*vr*II-sensitive exon 22-containing Ca_V_1.2 isoform is not expressed, while under the pathophysiological proliferating state, the exon 21 is completely switched to exon 22. Based on these findings, inhibition of the exon 22 inclusion level may suppress the smooth muscle cell proliferation that leads to vascular remodeling in atherosclerosis.

### 4.4. Hypertension

Hypertension is a leading cause of cardiovascular diseases such as coronary heart diseases and stroke [48]. Although the upregulated protein level of the smooth muscle Ca_V_1.2 channel is essential for hypertension [1,29], the underlying mechanisms are still unclear. Given the diversified pharmacological and biophysical properties, various vascular Ca_V_1.2 splice isoforms have been reported to associate with hypertension. Generally, the alternative splicing of *CACNA1C* in smooth muscle is limited to three mutually exclusive (1b/c, 21/22 and 31/32) and two alternate (9*, 33) exons [49].

In 4.5-month-old spontaneously hypertensive rats (SHR), the exon 9* (RGSC 6.0/rn6, chr4: 150760674-150760748) inclusion level in the left ventricle was increased from 3% to 11% in Wistar Kyoto (WKY) rats [21], and was upregulated from 40.1% to 50.4% in mesenteric artery [50]. More importantly, exon 9*-containing Ca_V_1.2 channels (Ca_V_1.2b, Figure 3) were considered to play a dominant role in the constriction of cerebral artery as the antisense oligonucleotides targeting Ca_V_1.2b channels led to larger constriction in intact cerebral arteries from New Zealand white rabbits (Broad/oryCun2, chr8: 35146587-35146660) compared to that of Ca_V_1.2_Δe9*_ channels (Figure 3) [51]. Additionally, whole-cell patch clamp recordings in transfected HEK 293 demonstrated that smooth muscle Ca_V_1.2b channels displayed hyperpolarized shift in voltage-dependent activation and *I–V* relationships by 9 and 11 mV, respectively, compared to Ca_V_1.2_Δe9*_ channels [52]. These results suggested an important role of exon 9* in contributing to the increased vasoconstriction in SHR vessels.

As for alternative splicing of exon 1 in smooth muscle, exon 1b (usually referred to as exon 1, RGSC 6.0/rn6, chr4: 151176108-151176156) is the most dominant isoform compared to exon 1c (RGSC 6.0/rn6, chr4: 151146677-151147042) with a ratio at 60:1 in mesenteric arteries from both WKY rats and SHR [53]. The protein level of exon 1b-containing Ca_V_1.2 channels (Ca_V_1.2_e1b_) showed a 3–4 fold increase in aorta and mesenteric artery from SHR compared to WKY rats by using an exon 1b-specific antibody [54]. However, the changes of Ca_V_1.2_e1c_ protein level remain to be determined although the mRNA level of exon 1c showed differences in between SHR and WKY rats, as the differences in mRNA levels are insufficient to account for the differences in Ca_V_1.2 protein levels in SHR.

## 5. Splice Variant-Specific Modulation of Ca_V_1.2 Channel Function

Alternative splicing diversifies Ca_V_1.2 function through inclusion or exclusion of alternative exons in various combinations. The combinatorial arrays of alternative exons may affect the modulation of Ca_V_1.2 channels by their interacting protein partners, such as Galectin-1 and α_2_δ-1 subunit, or may affect their sensitivity to calcium channel blockers, such as DHPs.

### 5.1. Modulation by Ca_V_1.2-Interacting Partners

Galectin-1, a member of the β-galactoside-binding protein family [55], was reported to bind to exon 9 within Ca_V_1.2 I–II loop and negatively modulate Ca_V_1.2 channel function [56]. However, inclusion of exon 9* downstream of exon 9 completely abolished the inhibitory effects of Galectin-1 protein on Ca_V_1.2 channel function [56], suggesting that the inhibitory effects of Galectin-1 is selective to Ca_V_1.2_Δe9*_ channels. This may be explained by the possibility that the ER export signals that contain a few negatively charged amino residues may interact with the positively charged amino acids found in the neighboring exon 9*, and hence this interaction prevents Galectin-1 binding and modulation.

The α_2_δ-1-subunit is one α_2_δ isoform that is largely expressed in skeletal muscle and is also present in cardiac and smooth muscle [57]. Notably, the α_2_δ-1-subunit is significantly elevated in cerebral arteries from SHR [13,14], and was found to selectively traffic exon 1c-containing Ca_V_1.2 channels (Ca_V_1.2_e1c_, Figure 3) to the plasma membrane of the cerebral artery [49]. Knock-down of α_2_δ-1 subunit inhibited the surface expression of Ca_V_1.2_e1c_ channels more than Ca_V_1.2_e1b_ channels. Also, as compared to Ca_V_1.2_e1b_ channels, knock-down of Ca_V_1.2_e1c_ channels by a short hairpin RNA (shRNA) resulted in a larger reduction of Ca_V_1.2 currents and vasodilation of cerebral artery. This study also suggested that the Ca_V_1.2 N-terminus may be a critical element required for channel trafficking in cerebral artery [49].

### 5.2. Modulation by Calcium Channel Blockers

Besides exon 8/8a, the exon 33 level in Ca_V_1.2 channels also affect the sensitivity to nifedipine, one of the first generation DHPs. In exon 33^−/−^ cardiomyocytes, the IC_50_ for nifedipine inhibition of the Ca_V_1.2_Δe33_ channels was 10.6 nM, as compared to 22.8 nM for Ca_V_1.2_e33_ in wild-type cardiomyocytes [45]. This study also strengthened the hypothesis that alternative splicing-induced changes of biophysical properties of Ca_V_1.2 channels are associated with the sensitivity to nifedipine blockade [4].

Diltiazem, a non-dihydropyridine calcium channel blocker, also displayed different inhibitory effects on the cardiac isoform (Ca_V_1.2a, Figure 3) and smooth muscle isoforms (Ca_V_1.2b and Ca_V_1.2_SM_, Figure 3) of Ca_V_1.2 channels [58]. The IC_50_ of diltiazem for the cardiac isoform of Cav1.2 was about two times that for the other two vascular smooth muscle isoforms. By substitution of cardiac exon 1a and/or 8a into smooth muscle exon 1 or 8, the IC_50_ of diltiazem for the chimeric cardiac isoform of Ca_V_1.2 channels was significantly reduced, suggesting that all three exons 1, 8 and 9* contribute to the different sensitivities of the cardiac and smooth muscle splice isoforms to diltiazem.

## 6. Regulation of Ca_V_1.2 Splicing by Splicing Factors

Based on the above findings, alternative splicing generated functionally distinct Ca_V_1.2 channels that may be involved in the pathology of or adaptation to cardiovascular diseases. Thus, it is essential to understand the upstream regulatory principles and cofactors which control the inclusion or exclusion of alternatively spliced exons. To date, there are three splice factors that were found to regulate Ca_V_1.2 splicing as follows.

### 6.1. Rbfox Proteins

The RNA-binding Fox family (Rbfox) proteins including Rbfox1 and Rbfox2 were reported to differentially regulate *CACNA1C* exon 9* and exon 33 expression in the mouse cortex during development [59]. Both Rbfox1 and Rbfox2 were induced and were able to bind to the adjacent introns of exon 9* and exon 33, thereby upregulating the exon 9* exclusion and exon 33 inclusion, respectively, during neuronal development. Similarly, Rbfox1 protein was also upregulated during postnatal maturation of zebrafish and murine hearts, but was found to be significantly decreased in hearts from patients with dilated cardiomyopathy [60] and from mice subject to transverse aortic constriction (TAC) [61]. Given the increased inclusion of exon 33 in human failing hearts [45], it suggests that the regulatory mechanisms underlying exon 33 inclusion by Rbfox1 may be different between cortical neurons and cardiomyocytes. More importantly, loss of Rbfox1 significantly contributed to the development of cardiac hypertrophy and heart failure in the mouse pressure-overload model and restored Rbfox1 expression was able to prevent pathological hypertrophy [61]. This study provides evidence that regulation of RNA splicing by Rbfox1 may play an important role in transcriptome reprogramming, including *CACNA1C* mRNA, during cardiac hypertrophy that influences the pathogenesis of the disease.

In addition to cardiac diseases, dysregulated Rbfox2 was recently reported to be involved in hypertension through regulating the splicing of *CACNA1C* exon 9* and exon 33 in arteries [50]. The total Rbfox2 protein level was increased by about three-fold in mesenteric arteries (MA) from SHR compared to WKY rats. However, the mRNA level of wild-type Rbfox2 was significantly downregulated in MA from SHR, while a dominant-negative form of Rbfox2 lacking exon 6 was markedly up-regulated, which eventually resulted in an increase of exon 9* by 10.3% and a decrease of exon 33 by 10.5% in MA from SHR.

### 6.2. PTBP1

Additionally, the polypyrimidine tract-binding protein (PTBP1) has been shown to strongly repress exon 8a (GRCm38/mm10, chr6: 118742265-118742368) inclusion and switch Ca_V_1.2 splicing to the exon 8 (GRCm38/mm10, chr6: 118741871-118741974) isoform through directly binding to the conserved sequence elements upstream of exon 8a in mouse cortex [62]. Inclusion of exon 8a was largely inhibited in mouse embryonic brains, but was found to be gradually upregulated during neuronal development in correlation with the depletion of PTBP1 [49]. Similarly, PTBP1 protein was highly expressed in embryonic hearts and then dramatically reduced during cardiac development in both rats and mice [63]. This may contribute to the dominant expression of exon 8a-containing Ca_V_1.2 channels in the heart [25]. However, more experiments are necessary to further validate the regulation of alternative splicing of exons 8/8a by PTBP1 in the heart.

### 6.3. RBM20

RNA-binding motif protein 20 (RBM20) is a well-known Titin splicing repressor [64] and a gene for hereditary cardiomyopathy [65,66]. Recently, deep sequencing of the cardiac transcriptome of rat and human validated RBM20-dependent regulation of Ca_V_1.2 splicing. RBM20 mainly regulated the splicing of exon 8, 9*, 22 and 31 in rat and human hearts. However, the RBM20-dependent variance at the inclusion level of these exons was quite weak as the score of change in percentage spliced-in (PSI) was less than 10 [66]. Hence, the cardiac effects of RBM20-mediated Ca_V_1.2 splicing remain to be determined.

Altogether, targeting splicing factors of *CACNA1C* may provide alternative ways to prevent or even treat cardiac diseases.

## 7. Conclusions

The L-type Ca_V_1.2 channel is the major pathway for Ca^2+^ influx to initiate contraction in cardiac and smooth muscles. Alternative splicing provides the fine-tuning of Ca_V_1.2 function in adaption to various cellular or tissue conditions or in response to various diseases. Understanding the splicing patterns of *CACNA1C* in diseases may not only help us evaluate the functional changes of Ca_V_1.2 channels, but also provide potential therapeutic targets for developing novel methods to manage cardiovascular diseases.
